# Epilithic Bacterial Assemblages on Subtidal Rocky Reefs: Variation Among Alternative Habitats at Ambient and Enhanced Nutrient Levels

**DOI:** 10.1007/s00248-023-02174-1

**Published:** 2023-02-15

**Authors:** Joseph Elsherbini, Christopher Corzett, Chiara Ravaglioli, Laura Tamburello, Martin Polz, Fabio Bulleri

**Affiliations:** 1 MIT Microbiology Graduate Program, Department of Civil and Environmental Engineering, Massachusetts Institute of Technology, Cambridge, MA 02138 USA; 2 Molecular and Computational Biology Section, Department of Biological Sciences, University of Southern California, Los Angeles, CA 90089 USA; 3 Dipartimento di Biologia, Università di Pisa, CoNISMa, Via Derna 1, 56126 Pisa, Italy; 4 Department of Integrative Marine Ecology, Ischia Marine Centre, Stazione Zoologica Anton Dohrn, 80077 Punta San Pietro, Ischia, (Naples) Italy; 5 Centre for Microbiology and Environmental Systems Science, Djerassiplatz 1, 1130 Vienna, Austria

**Keywords:** Temperate rocky reefs, Epilithic microbial biofilms, Marine forests, Algal turfs, Urchin barrens

## Abstract

Temperate rocky reefs often support mosaics of alternative habitats such as macroalgal forests, algal turfs and sea urchin barrens. Although the composition of epilithic microbial biofilms (EMBs) is recognized as a major determinant of macroalgal recruitment, their role in regulating the stability of alternative habitats on temperate rocky reefs remains unexplored. On shallow rocky reefs of the Island of Capraia (NW Mediterranean), we compared EMB structure among canopy stands formed by the fucoid *Ericaria brachycarpa*, algal turfs, and urchin barrens under ambient versus experimentally enhanced nutrient levels. The three habitats shared a core microbial community consisting of 21.6 and 25.3% of total ASVs under ambient and enhanced nutrient conditions, respectively. Although Gammaproteobacteria, Alphaproteobacteria and Flavobacteriia were the most abundant classes across habitats, multivariate analyses at the ASV level showed marked differences in EMB composition among habitats. Enhancing nutrient level had no significant effect on EMBs, although it increased their similarity between macroalgal canopy and turf habitats. At both ambient and enriched nutrient levels, ASVs mostly belonging to Proteobacteria and Bacteroidetes were more abundant in EMBs from macroalgal canopies than barrens. In contrast, ASVs belonging to the phylum of Proteobacteria and, in particular, to the families of Rhodobacteraceae and Flavobacteriaceae at ambient nutrient levels and of Rhodobacteraceae and Bacteriovoracaceae at enhanced nutrient levels were more abundant in turf than canopy habitats. Our results show that primary surfaces from alternative habitats that form mosaics on shallow rocky reefs in oligotrophic areas host distinct microbial communities that are, to some extent, resistant to moderate nutrient enhancement. Understanding the role of EMBs in generating reinforcing feedback under different nutrient loading regimes appears crucial to advance our understanding of the mechanisms underpinning the stability of habitats alternative to macroalgal forests as well as their role in regulating reverse shifts.

**Supplementary Information:**

The online version contains supplementary material available at 10.1007/s00248-023-02174-1.

## Introduction 

Human activities are causing unprecedented changes to terrestrial and aquatic systems, impairing their functioning and ability to deliver services globally [1, 2]. Alterations in biotic or abiotic conditions can shift ecosystems to alternative undesirable states, which are generally characterized by lower species diversity and productivity [3–5]. Once the transition has occurred, stabilizing feedbacks can lock the system into the degraded state [3, 5–9]. Thus, understanding the mechanisms generating hysteresis is key for conserving the resilience of pristine systems and for promoting reverse shifts in degraded systems.

Subtidal macroalgal forests, composed of Laminariales and Fucales, support biodiversity through the formation of habitat and are among the most productive marine systems [9–11], delivering key ecosystem services to humans [9, 12]. Nonetheless, the collapse of these systems has been documented along temperate coasts worldwide [10, 13–15]. Overgrazing by either sea urchins or fish can cause the shift in rocky reef dominance from canopy forming (i.e., macroalgal forests) to encrusting coralline macroalgae (i.e., sea urchin barrens), while excessive nutrient loading can elicit algal turf dominance [5, 6, 9, 14–16]. Processes that reduce the recruitment of canopy-forming macroalgae contribute to barren or algal turf stabilization [6]. Continuous scraping by sea urchins, fish or limpets and reduced supply of spores or gametes from surrounding reproductive plants underpin reduced recruitment in barren areas [6, 17–19]. Conversely, accumulation and trapping of sediments in the complex three-dimensional matrix formed by algal turfs reduce the settlement of spores or zygotes [10, 20–22]. Although the composition of epilithic microbial biofilms (EMBs) is recognized as a major determinant of macroalgal recruitment through the modification of physical and chemical characteristics of settlement surfaces [23, 24], their relevance in sustaining the stability of different states on temperate rocky reefs remains unexplored.

Brown seaweeds belonging to the genus *Cystoseira sensu lato* are important habitat-formers along Mediterranean rocky coasts, but they are declining throughout the basin as a consequence of human alterations of abiotic (i.e., enhanced sedimentation rates and nutrient loading) and biotic conditions (i.e., increased herbivory due to predator over-exploitation) [15, 25–28]. Indeed, even at relatively pristine sites, macroalgal canopy stands often alternate with areas occupied by algal turfs or encrusting corallines [28]. However, to the best of our knowledge, no study has attempted to compare EMBs among alternative habitats that form mosaics on rocky reefs. Although, according to the evidence from coral reefs [29–31], EMBs from canopy stands, algal turfs and urchin barrens can be expected to differ, we have no clue of the magnitude of these differences.

Macroalgae can condition bacterial biofilms on underlying or adjacent hard surfaces through different mechanisms. For instance, macroalgal release of dissolved organic carbon (DOC), rich in labile sugars, has been shown to promote the growth of copiotrophic bacteria on coral reefs [32] and to differ among macroalgal species [33]. Likewise, there is strong variation among macroalgal species in the type and amount of allelochemicals they produce and release in the water column [34]. Free-living microbes, exosomes, and allelochemicals can be released by macroalgae into the diffusive boundary layer (DBL) and transferred to neighboring surfaces [35–37]. Although large variation in the structure of epiphytic microalgal communities has been documented at a hierarchy of spatial scales [38], the composition of epiphytic bacterial communities on macroalgae is generally highly host-specific [39–42] and a core microbial community is often maintained [24, 43–45]. Different macroalgal species or assemblages can also cause variation in epilithic bacterial assemblages by influencing physical conditions, such as illumination and water-flow. For these reasons, the structure of epilithic biofilms can be expected to vary among areas dominated by different macroalgal assemblages. Thus, the aim of this study was to test the hypothesis that EMBs differ between canopy-dominated areas and alternative habitats (i.e., urchin barrens or algal turfs) on shallow rocky reefs.

In addition, since excessive nutrient loading due to run-off from urban and agricultural areas has been widely shown to promote macroalgal growth on both temperate and tropical reefs [8, 10] and, indeed, to be an important driver of the decline of temperate macroalgal forests [10, 16, 20], we also experimentally tested the hypothesis that variations in the epilithic microbial biofilms among alternative habitats are influenced by nutrient enrichment. Evidence from tropical reefs suggests, in fact, that nutrient levels contribute to shape the EMB composition [29, 30].

## Materials and Methods

This study was carried out on shallow rocky reefs at Capraia Island in the Tuscan Archipelago (NW Mediterranean) from June 2014 to July 2016. The island can be considered pristine since, due to the very small resident population, lack of relevant agricultural activities and distance from mainland (~ 30 nautical miles), it is not exposed to inorganic and organic pollution or nutrient run-off [28]. Rocky reefs between 2 and 8 m deep are dominated by canopies stands formed by the fucoid *Ericaria brachycarpa*, which alternates with patches of algal turfs or urchin barrens colonized by encrusting corallines [28]. Here, algal turfs are composed of foliose (e.g., *Dictyota* spp., *Padina pavonica*), filamentous (e.g., Sphacelariales), siphonous algae (e.g., *Acetabularia acetabulum*, *Caulerpa cylindracea*), and corticated rhodophytae (e.g., *Laurencia obtusa*, *Gastroclonium* sp.).

**Fig. 1:**
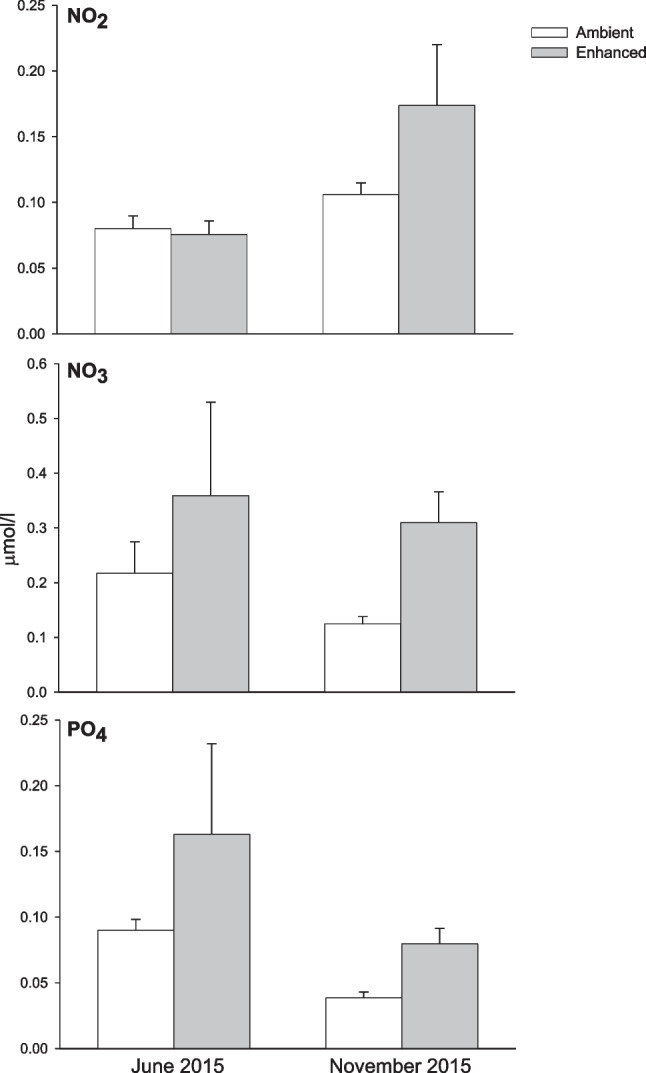
Nitrite, nitrate, and phosphate concentrations in seawater collected from enriched versus control areas in June and November 2015 (means ± SE; *n* = 12)

In June 2014, four 1.5 × 0.5 m areas of each habitat (macroalgal canopies, algal turfs, and urchin barrens) were randomly identified on large boulders at 4–6 m depth and about 10 m apart, along a 600-m stretch of coast (a total of 12 areas). Barren areas were adjacent to canopy stands. Two areas of each habitat were exposed to nutrient addition and two areas were left at ambient nutrient conditions (i.e., controls). Nutrients were elevated by means of slow-release fertilizer pellets (Osmocote®, 6 months, 17:11:10 N:P:K) contained in plastic net bags (1-mm mesh size), a common procedure on rocky reefs [46–49]. Nutrient enrichment was designed to simulate conditions comparable to those recorded in urban areas of the same region [50]. Eight mesh bags, containing about 100 g of fertilizer each, were fixed in each area assigned to nutrient enrichment by means of steel hooks anchored to the substratum. Nutrient bags were replaced every 3 months throughout the duration of the study in order to maintain nutrient release [47]. This method has been successfully used on temperate reefs, since it allows to generate a localized nutrient enrichment [47, 49–51]. In order to assess nutrient concentrations in experimental areas, seawater samples were collected from each area at two random times, in June and November 2015. Two samples taken approximately 3 cm above each area using a 60-ml syringe were immediately filtered (0.45 μm) and frozen for transportation to the laboratory. Nutrient concentrations were quantified by means of a continuous-flow AA3 Auto-Analyzer (Bran-Luebbe), according to Grasshoff et al. [52]. Each sample was analyzed in technical triplicates to obtain an average value. Our study area is oligotrophic [28] and the experimental nutrient enrichment was effective in enhancing nitrate, nitrite, and phosphate concentrations (Fig. 1), although to levels lower than those recorded in areas exposed to intense human activities [50, 53]. Dissolution rates of fertilizer pellets vary with temperature and on fine spatial and temporal scales according to hydrodynamic regimes, making it difficult to obtain precise estimates of nutrient concentrations through the analysis of water samples [47, 49, 54]. Thus, water sample analyses were complemented by quantifying the rate of pellet dissolution, as suggested by Carnell and Keough [48]. The weight of the fertilizer in each nutrient bag was measured at the third decimal by means of a precision scale before deployment. Upon retrieval, nutrient bags were dried in a muffle for 28 h at 60 °C, and the amount of fertilizer that had not dissolved was re-weighted in order to estimate the average nutrient release rate per day over the duration of the experiment [48]. Nutrient pellets dissolved at an average rate of 2.3 g per day per area (data averaged across 5 sampling times: October 2014 and March, June, August, and November 2015).

Since budgetary constraints prevented multiple samplings, we assessed the response of EMBs to fertilization after 2 years since the start of the experiment (i.e., in July 2016), a period of time generally sufficient to trigger changes in the benthic communities within each habitat including canopy-stands [46, 55, 56]. Using hammer and chisel, 3 chips of rock about 3 cm^2^ and free of erect macroalgae and sessile invertebrates were collected within each study area and were inserted into sterile 50 ml centrifuge tubes and immediately placed on ice. To harvest epilithic bacterial assemblages, chips were washed twice with 40 ml filter-sterilized artificial seawater (ASW) made with Sea Salts (Sigma S9883) to remove seawater carryover, resuspended in 40 ml ASW, and then vigorously shaken for 30 s to dislodge and suspend surface-associated bacterial communities. The resulting supernatant was then transferred to a sterile lur-lock syringe and filtered into a 0.22-µm Sterivex filter (Millipore SVGV010RS). Filter cartridges were then stored in sterile bags and immediately placed on ice prior to long-term storage at – 80 °C.

DNA was extracted from samples using a modified method adapted from Thompson et al. [57]. Sterivex filters were removed from the casing and excised using a sterile razor blade and placed in a 2-ml screw cap microfuge tube with 0.2 g of 0.1 mm zirconium beads (Biospec Products) with 750 ml cell lysis solution (PureGene Cell and Tissue Kit, Gentra Systems). Filters were bead beated at 5,000 rpm for 1 min, placed at 80 °C for 5 min, and then stored at − 20 °C until further processing. DNA extraction was initiated by first incubating samples with 4 ul Rnase at 37 °C for 30 min, followed by addition of 250 ul protein precipitation solution (PureGene Cell and Tissue Kit; Gentra Systems). Samples were then centrifuged for 3 min at 15,000 × g to remove filter debris, beads, and precipitated protein before transferring the supernatant to a clean 1.5-ml Eppendorf tube. Samples were spun again at 15,000 × g for 3 min, and the supernatant was placed into a clean 1.5-ml Eppendorf tube containing 750 ul 100% IPA and pipette mixed. Samples were centrifuged for 1 min at 15,000 × g, and precipitated DNA was washed twice with 75 and 70% ethanol solution, air dried for 30 min, resuspended in 100 ul DNA hydration solution (PureGene Cell and Tissue Kit; Gentra Systems), and stored at – 20 °C until community libraries were constructed. DNA could not be extracted from one sample taken in an urchin barren area. Amplicon libraries were prepared using methods described in [58]. Briefly, the 16S V4 region was amplified using the primers 515F (GTGCCAGCMGCCGCGGTAA) and 806R (GGACTACHVGGGTWTCTAAT) for 13 cycles, and then in Illumina, adapters and barcodes were added in subsequent steps. These libraries were sequenced using Illumina Miseq 2 × 250 paired end at the BioMicro Center (Massachusetts Institute of Technology, Cambridge, MA). The primers were removed from forward and reverse reads using a custom python script, allowing one mismatch and discarding reads without the primer. The reads were then quality filtered and truncated to a common length, allowing for up to two expected errors and truncating to 140 bases for the forward reads and 140 for the reverse. Dada2 [59] was used to infer the Amplicon Sequence Variants (ASVs) from the raw reads that have been deposited in the National Center for Biotechnology Information (NCBI) under study accession number PRJNA901976 (https://www.ncbi.nlm.nih.gov/bioproject/PRJNA901976). Pseudopooling was performed by running Dada2 twice and using all identified ASVs from the first run as prior sequences in the second, as described on the pooling documentation on the Dada2 website. This reduces the risk of false negatives with Dada2, where sequences with only one or two reads that are close to a more abundant sequence are erroneously corrected. After denoizing and merging, the ASVs were assigned using a naive Bayes classifier implemented in Dada2 against the RDP 16S rRNA gene database [60].

## Statistical Analyses

Variations in the structure of epilithic microbial biofilms (EMBs) among habitats and nutrient levels were assessed by means of PERMANOVAs including the factors Habitat (fixed, with 3 levels, canopy versus turf versus barren), Nutrients (fixed, with 2 levels, ambient versus enhanced), and Area (random, 2 levels and nested within the interaction habitat × nutrients) on pairwise Euclidean distances of centered and log-ratio transformed (CLR) relative ASV abundances. Multivariate patterns were visualized in two dimensions using nonmetric multidimensional scaling (nMDS). These multivariate analyses were run using the software Primer and Permanova + v.1.0.1 (PRIMER-e). In addition, normalized pairwise distances (where 1.0 represents the maximum observed Euclidean distance) were visualized using the R package Vegan vers. 2.5–4 [61] in order to assess how nutrient addition influenced differences in microbial communities between either algal turfs or sea urchin barrens and *E. brachycarpa* canopies. In addition, Venn diagrams were built using the software Venny 2.1 [62] to identify the microbial core community on rocky reefs under ambient and enhanced nutrient conditions.

For each ASV, two-sided *t*-tests were performed to compare their relative abundance between macroalgal canopies and either turfs or urchin barrens at natural or enhanced nutrient levels using base functions in R 3.6.0 [63]. The Holm–Bonferroni method was used to deal with family-wise error rates for multiple hypothesis tests.

## Results

**Fig. 2:**
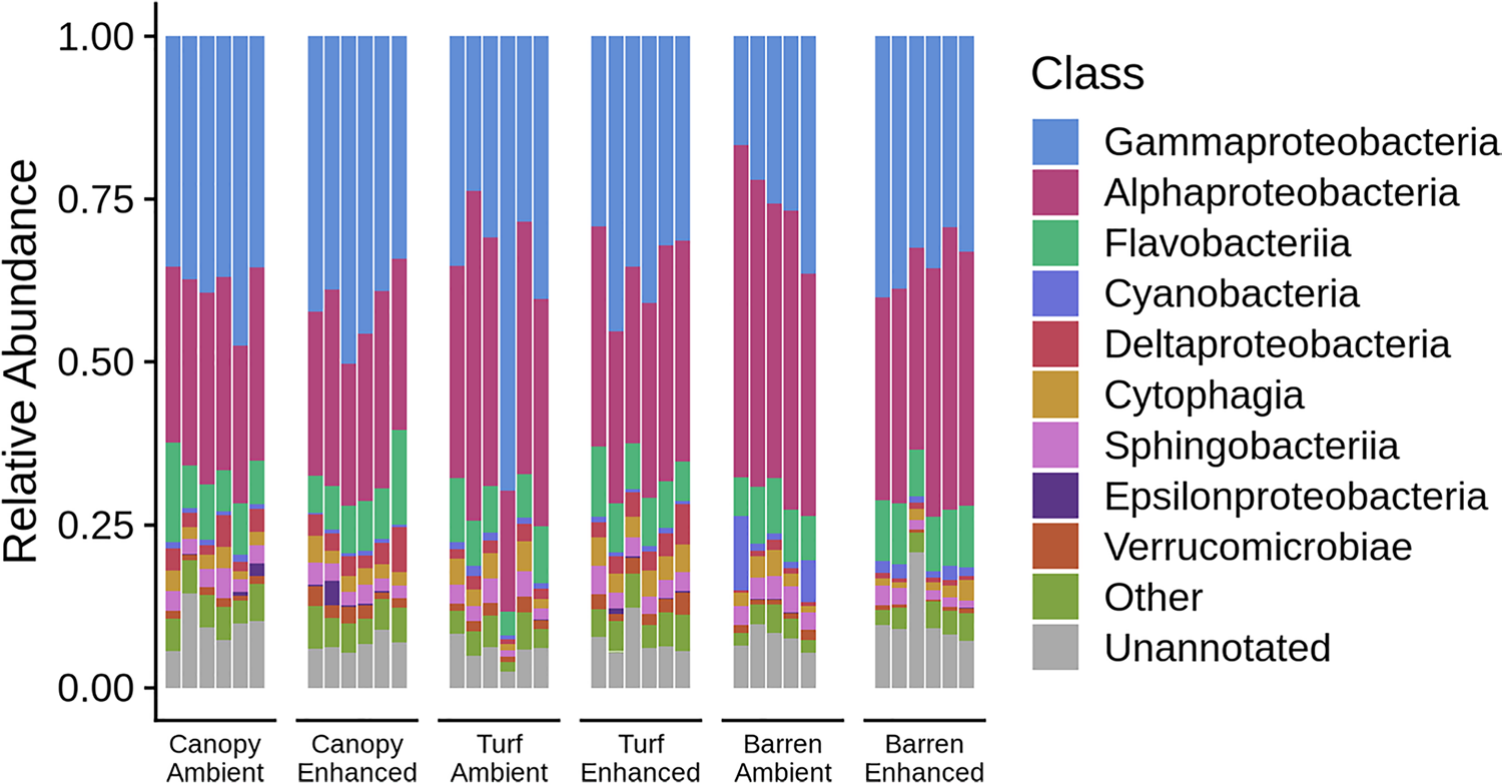
Taxonomic composition of epilithic microbial biofilms at the class level

**Fig. 3:**
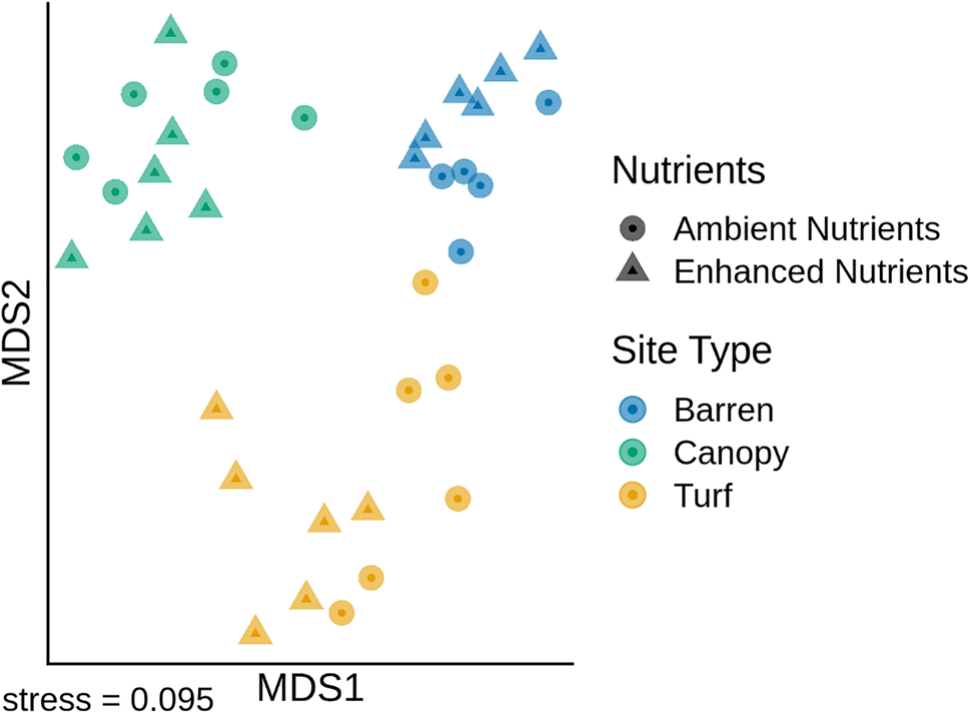
Two-dimensional nMDS on Euclidean distances calculated from untransformed data comparing the structure of epilithic microbial biofilms among habitats (macroalgal canopies versus urchin barrens versus algal turfs) at ambient and enriched nutrient levels

**Fig. 4:**
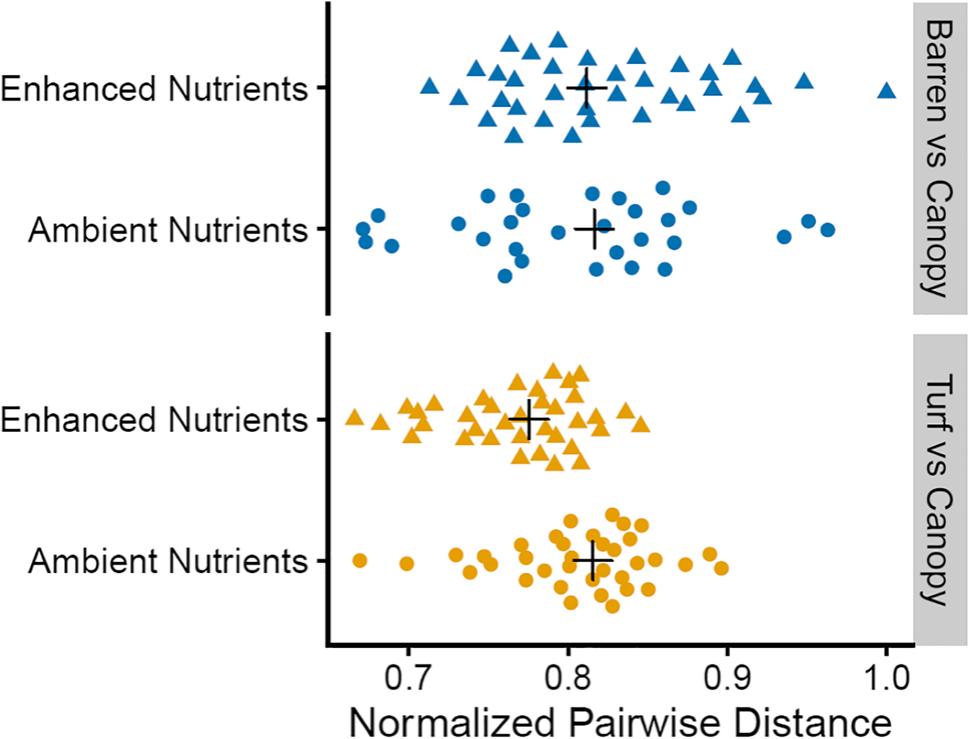
The normalized pairwise distance (where 1.0 represents the maximum observed Euclidean distance) visualized for the urchin barren and algal turf communities with and without added nutrients compared to the macroalgal canopy communities

Gammaproteobacteria, Alphaproteobacteria and Flavobacteriia were the most abundant classes across habitats at both ambient and enhanced nutrient levels and no consistent trends in variation among the treatments at this high taxonomic level were evident (Fig. 2). However, when using the highest taxonomic resolution afforded by the 16S rRNA gene tag sequence, the ASV level, compositional differences emerged. The PERMANOVA on Euclidean distances and post hoc comparisons showed that the structure of bacterial assemblages differed among habitats (Table 1) (Fig. 3). In contrast, nutrient enrichment had no significant effect on bacterial assemblages. However, bacteria in turf areas appeared more variable than those in canopy or barren areas and showed larger changes in response to nutrients (Fig. 3). Following the addition of nutrients, the microbial assemblage in turf habitats became more similar to that underneath canopies. In contrast, nutrient addition to the barren areas did not shift the microbial community toward that found in canopy-dominated areas (Fig. 4).

**Table 1:** PERMANOVA comparing the structure of EMBs among habitats (macroalgal canopies versus urchin barrens versus algal turfs), between nutrient levels (ambient versus enriched) and between areas. Pairwise comparisons are reported for significant factors

Source of variation	df	MS	Pseudo-*F*	*P *(perm)
Habitat = H	2	39,273.0	7.114	0.001
Nutrients = N	1	9320.2	1.689	0.086
H × N	2	7055.3	1.278	0.135
Area (H × N)	6	5530.0	1.477	0.001
Residual	23	3743.8		
Pairwise comparisons				
Habitat	t	*P *(perm)		
Turf–barren	2.558	0.026		
Turf–canopy	2.277	0.033		
Barren–canopy	3.277	0.030

**Fig. 5:**
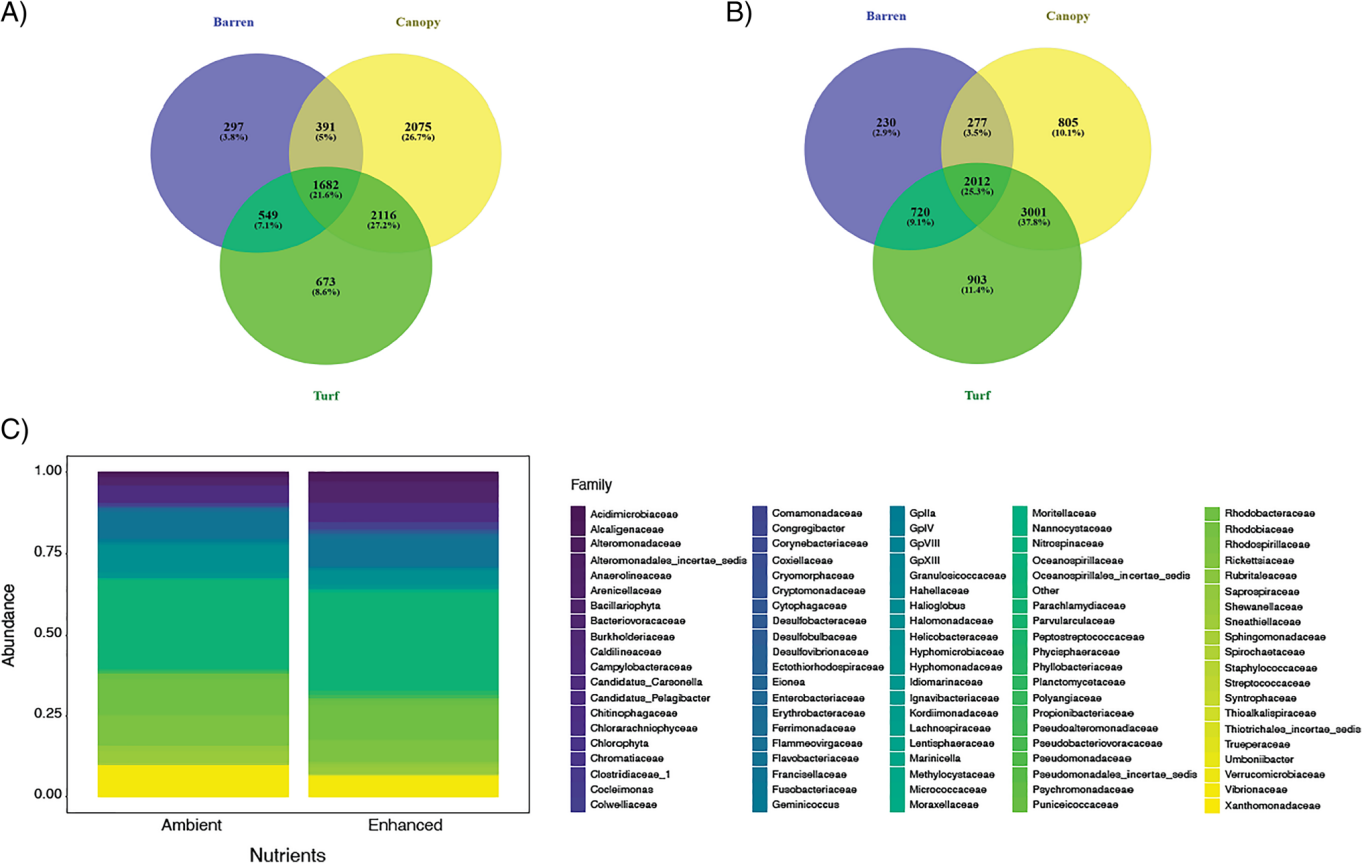
Venn diagrams showing the percentage of total ASVs shared across the three habitats (i.e., the core microbiome) and between each pair of habitats, at **A** ambient versus **B** enhanced nutrient levels. Stacked barplots (**C**) illustrate the composition of the core microbiome, at the family level, separately for ambient and enhanced nutrient levels

The three habitats shared a core microbial community consisting of 21.6 and 25.3% of ASVs under ambient and enhanced nutrient conditions, respectively (Fig. 5A and B). The addition of nutrients increased the percentage of ASVs shared between canopy- and turf-dominated habitats from 48.8 to 63.1%. In contrast, there were no major changes in the percentage of common ASVs between barrens and the other habitats at ambient versus enhanced nutrient conditions. Under ambient nutrients, the core microbial community of the rocky reef was mostly composed of the families of Candidatus_Pelagibacter (~ 5.3%), Flavobacteriaceae (~ 7.9%), Halomonadaceae (~ 8.2%), Rhodobacteraceae (~ 11.2%), Rhodospirillaceae (~ 9.2%) and Vibrionaceae (~ 9.6%), which, together, accounted for ~ 51.4% of total ASVs (Fig. 5C). The same families (Candidatus_Pelagibacter: ~ 5.3%, Flavobacteriaceae: ~ 7.9%, Halomonadaceae: ~ 4.1%, Rhodobacteraceae: ~ 10.6%, Rhodospirillaceae: ~ 6.7% and Vibrionaceae: ~ 6.4%), in addition to Bacillariophyta (~ 5.7%), were also the most represented under enhanced nutrient conditions. ASVs non-identified to family level accounted for ~ 27.2 and ~ 30.0% of the total ASV number at ambient and enhanced nutrient level, respectively.

**Fig. 6:**
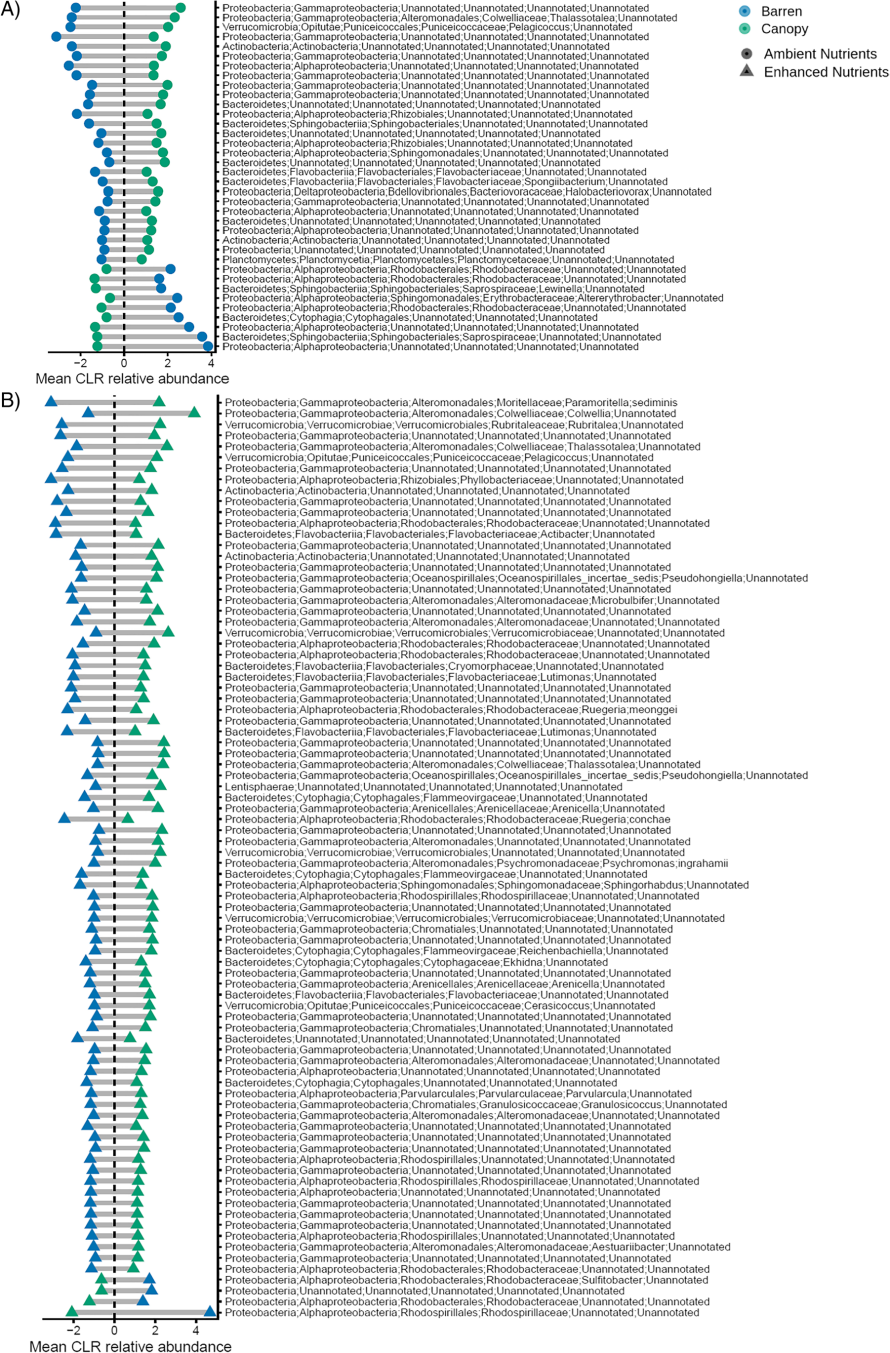
Mean CLR-transformed abundance of ASV differing significantly between macroalgal canopy and urchin barren habitats at **A** ambient and **B** enhanced nutrient levels

At ambient nutrient levels, the relative abundance of 38 ASV, most belonging to the phyla of Bacteroidetes (~ 31%) and Proteobacteria (~ 58%), differed significantly between barren and canopy areas (Fig. 6A; Online Resource 1). Most of the ASVs (i.e., ~ 74%) had a greater relative abundance in canopy than barren habitats. A higher number of ASVs (i.e., 84) differed between barren and canopy areas exposed to nutrient addition (Fig. 6B; Online Resource 2). Again, most of the ASVs (~ 95%) differentiating between these two habitats had a greater abundance in canopy than barren habitats, and only seven of them were important differentiators between barren and canopy areas also at ambient nutrients. Many ASVs could not be classified at the genus or species level. In all samples, > 50% of the relative abundance was accounted for by ASVs classified to the least order, and > 50% of the relative abundance was not assignable at the genus level (Fig. S1 in Online Resource 3).

**Fig. 7:**
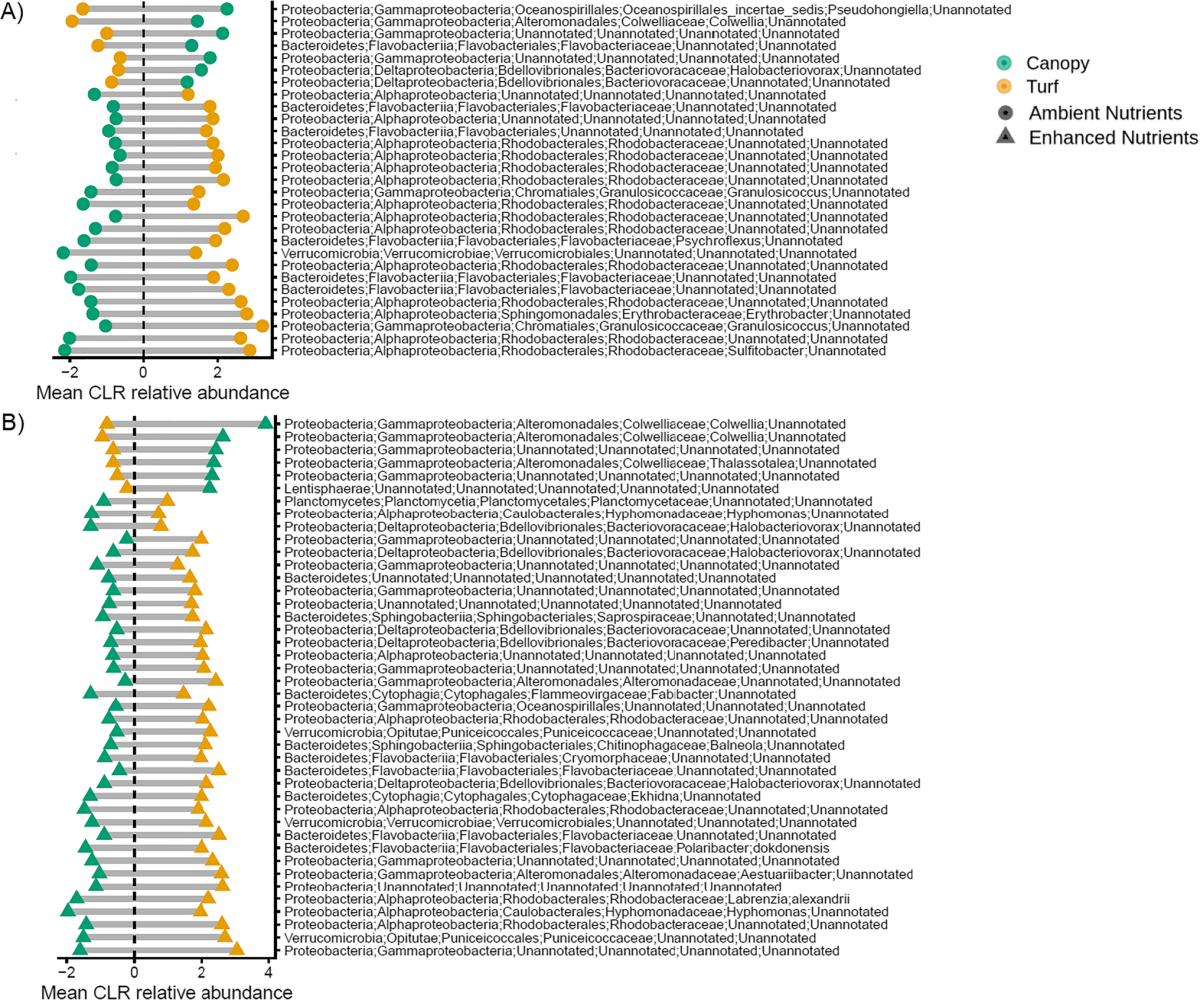
Mean CLR-transformed abundance of ASV differing significantly between macroalgal canopy and algal turf habitats at **A** ambient and **B** enhanced nutrient levels

The relative abundance of 30 and 42 ASVs differed significantly between turf and canopy areas at ambient and enhanced nutrient levels, respectively (Fig. 7A and B; Online Resources 4 and 5). There was no ASV in common between the groups that differentiated between these habitats at ambient versus enhanced nutrient levels. At both ambient and enhanced nutrient levels, differences between canopy and turfs were due to a large number of ASVs (~ 73 and ~ 86% of those differing between habitats respectively) being more abundant in turf habitats. Twenty-two and 36 ASVs, mostly belonging to the phylum of Proteobacteria and, in particular, to the families of Rhodobacteraceae and Flavobacteriaceae at ambient nutrient levels and of Rhodobacteraceae and Bacteriovoracaceae at enhanced nutrient levels were more abundant in turf habitats. Only 8 ASVs, some belonging to the families of Flavobacteriaceae, Colwelliaceae, Bacteriovoracaceae, Oceanospirillales incertae sedis and GpVIII Cyanobacteria, were more abundant in canopy habitats at ambient nutrient levels. Likewise, only 6 ASVs, three of which belonged to the family of Colwelliaceae, were enriched in canopy habitats exposed to enhanced nutrient levels.

## Discussion

The collapse of marine forests and their replacement by either encrusting coralline barrens or algal turfs are a global phenomenon [5, 10, 14, 64], widely documented along the coasts of the Mediterranean Sea [18, 19, 65]. Thus, understanding the mechanisms underpinning the stability of habitats alternative to forests appears key to devise sound strategies for sustaining the biodiversity and functioning of temperate rocky reefs.

A correlation between the composition of benthic organisms (i.e., algae and invertebrates) and microbial communities has been documented on coral reefs [29–31]. Although there is some evidence of species-specific influence of macroalgae on seawater [66, 67] and hard substrata microbial communities [68–70], to the best of our knowledge, this is the first study attempting to describe variation in epilithic microbial communities among the alternative habitats, i.e., macroalgal forests, urchin barrens and algal turfs, that compose mosaics on temperate rocky reefs. EMBs were mostly composed of Proteobacteria and Bacteroidetes, widely distributed and abundant bacterial phyla in the global oceans [71, 72] and reported as dominant on macroalgal surfaces, rocky substrata and marine sediments [68, 73, 74]. Nonetheless, when compared at the ASV level, EMBs from alternative habitats differed at both natural and enhanced nutrient levels, in accordance with previous studies that documented decreased similarity in microbial assemblages from higher to lower taxonomic levels [68]. Indeed, only about 22% of ASVs were common to the three habitats, suggesting that the core microbiome of this rocky reef was a small subset of the total number of ASVs recorded on the scale of the study site. Thus, the influence of small-scale habitat patchiness on Mediterranean shallow rocky reefs is not limited to eukaryotes, i.e., the associated fish and macroalgal and invertebrate assemblages [56, 75, 76], but extends to the prokaryotic compartment.

Several mechanisms can underpin EMB control by macroalgae, including the release of dissolved organic carbon and combined neutral sugars, secondary metabolites, exosomes, and free-living bacteria [35, 77–82]. In addition, changes in EMBs might be generated by macroalga-induced modification of environmental conditions such as light, water flow and sedimentation rates, and water chemistry, including pH and alkalinity [66]. Our study cannot provide a mechanistic explanation of differences in EMB composition among habitats, but together with evidence of macroalgal settlement being influenced by bacteria [23, 24, 42, 83], it suggests that epilithic microbial community control by dominant benthic macroalgae might represent a stabilizing feedback and, hence, a mechanism underpinning the stability of alternative stable states documented on temperate rocky reefs [5, 6].

Alphaproteobacteria, Gammaproteobacteria and, to a lesser extent, Flavobacteriia were dominant in EMBs, and variation in the relative abundance of ASVs belonging to these classes contributed most to differentiate between macroalgal canopies and either barrens or algal turfs. EMBs from sea urchin barrens and algal turfs were remarkably different from those occurring within macroalgal canopies. However, such differences were produced by different patterns of variation in ASV occurrence and relative abundance between canopy and either turf or barren habitats. A large proportion of ASVs differentiating EMBs between canopies and barrens had a higher relative abundance in the former habitat. In contrast, differences between forests and turfs were mostly due to turf enrichment in almost all of the differentiating ASVs. This may indicate that DOC release by macroalgae, reported to be higher in turfs [33], might have enhanced the growth of bacteria known to play a key role in algal C cycling, such as Rhodobacteraceae [84]. These patterns were even more pronounced under enhanced nutrient conditions, suggesting that release from N and P limitation, along with DOC release from macroalgae, could have fostered growth of ASVs hosted by primary surface beneath canopy and, especially, within turf-forming macroalgal assemblages.

Nonetheless, the experimental elevation of nutrient levels had no significant effect on the bacterial composition of EMBs. Our results are in contrast with those of Remple et al. [29], who documented nutrient-driven alteration in the structure of bacterial biofilms on coral reef substrata. It is, however, worth noting that enriched levels in our experiment were moderate (NO_3_^−^ = 0.319 ± 0.024 μmol L^−1^) and one order of magnitude lower than mid or high levels generated by Remple et al. (respectively, 2.68 and 6.64 μml L^−1^ NO_3_^−^).

Although the multivariate analysis did not show significant effects of nutrient enrichment, the response of EMBs from algal turfs appears somewhat stronger than that from macroalgal canopies and urchin barrens. In fact, normalized pairwise distances indicate that nutrient enrichment reduced the dissimilarity in EMBs structure between canopies and algal turfs but not barrens. Indeed, the proportion of ASVs shared between canopy- and turf-dominated habitats increased by about 15% under enhanced nutrient condition. These patterns might be connected with the response to nutrient enrichment of the main macroalgal species or functional groups that form each of the three habitats and, more specifically, to changes in their biomass. Inside forests, nutrient enrichment did not cause major changes in plant size and canopy cover and in the composition of their epiphyte assemblage [55, 56]. Large-sized brown seaweeds such as Fucales and Laminariales have an efficient external uptake of N and may have, thus, reduced the influence of the experimental nutrient enrichment on EMBs [55, 85]. Likewise, in urchin barrens, nutrient enrichment was unlikely to cause a major increase in the biomass of encrusting corallines due to morphological characteristics of their prostrate, calcareous thallus. The weak response of encrusting corallines to nutrients may also explain little changes in the proportion of ASVs shared between barrens and either macroalgal canopies or turfs in response to fertilization. EMBs from barren areas appear therefore more stable than those on substrata under the influence of algal turfs.

Unfortunately, we did not assess changes in the structure of turfing assemblages, but nutrient-induced increases in cover and biomass of algal turfs have been documented globally [20]. An increment in their biomass [55] may have influenced the EMBs through a greater release of DOC. This would indicate that variations in the biomass of primary producers would underpin the differences in the structure of EMBs they host. There is also evidence indicating that the amount of DOC released varies among macroalgal species and it is particularly elevated in algal turfs [33]. Indeed, copiotrophic bacteria, such as those belonging to the classes of Flavobacteriales and Rhodobacteriales, had a greater relative abundance in EMBs from algal turfs than macroalgal canopies and contributed to differentiate these two habitats at ambient nutrient levels.

While the number of ASVs differentiating between canopies and algal turfs did not change according to nutrient levels, those that differentiated between canopies and barrens doubled under nutrient enrichment. In both cases, differences were mainly driven by ASVs, most of which (~ 77%) belonging to the classes of Alphaproteobacteria or Gammaproteobacteria having a greater relative abundance in EMBs from macroalgal canopies than barrens. However, in both cases, there were ASVs which were markedly more represented in barrens, two unannotated belonging to Alphaproteobacteria and one to the family of Saprospiraceae under ambient nutrient conditions and one belonging to the family of Rhodospirillaceae under elevated nutrients. This might suggest that EMBs that colonize barren areas could be characterized by few dominant ASVs.

The influence of mono- or multi-specific bacterial assemblages on macroalgal recruitment has been examined for just a few species (e.g., *Ulva*, *Enteromorpha*, and *Polysiphonia*) [23] and there is no information on which bacterial taxa facilitate or hinder fucoid recruitment. Our results suggest that experimental assessment of the role of EMBs in regulating fucoid recruitment in barrens is warranted to deepen our understanding of the mechanisms that generate hysteresis in each of these alternative, stable habitats. While a great deal of research has attempted to assess how human-induced changes in microbiomes affect the phenology, physiology, and ecology of habitat-forming species [86], a smaller effort has been produced to assess the role of microbial biofilms in regulating the stability and persistence of habitats formed by alternative species. This is at odds with evidence from coral reefs that macroalga-induced modifications in the composition of epilithic microbial biofilms reduce coral recruitment, contributing to the stability of the macroalgal dominated state [29, 30, 69, 70]. Our study brings some evidence of major differences in the structure of EMBs from the alternative habitats that characterize shallow temperate rocky reefs and suggests that the characteristics of microbial biofilms that develop within algal turfs and urchin barrens may contribute to enhance their stability by limiting the settlement of canopy-forming species. Reducing herbivore pressure through the restoration of lost predatory interactions and controlling nutrient inputs from urban and agricultural settings have been identified as priorities for the conservation of marine forests and for enhancing their recovery [10, 87]. Nonetheless, these strategies may not be effective to trigger shifts back to the forested state due to hysteresis. Thus, experimental assessment of the ability of canopy-forming species to settle on surfaces colonized by EMBs that have been developed in the presence of adult conspecifics versus either algal turfs or urchin barrens appears warranted to enhance our understanding of the mechanisms that regulate shifts among alternative stable states on temperate rocky reefs. Finally, since the weak response of EMBs to fertilization could be due to the moderate nutrient enhancement generated in our study, experimental elevation of nutrients to levels matching those recorded in areas exposed to run-off from urban or agricultural areas is necessary to get a deeper insight into their effects on EMBs.

## Supplementary Information

Below is the link to the electronic supplementary material.

Supplementary file1 (XLSX 379 KB)

Supplementary file2 (XLSX 379 KB)

Supplementary file3 (DOCX 60 KB)

Supplementary file4 (XLSX 380 KB)

Supplementary file5 (XLSX 382 KB)

## Data Availability

The datasets generated and analysed during the current study are available from the corresponding author.
