# Supplementary material for: Epilithic Bacterial Assemblages on Subtidal Rocky Reefs: Variation Among Alternative Habitats at Ambient and Enhanced Nutrient Levels
Source: Microb Ecol. 2023 Feb 15;86(3):1552–64. doi: 10.1007/s00248-023-02174-1 (PMC10497455; doi:10.1007/s00248-023-02174-1)
Supplement: Supplementary file 3 — Supplementary file3 (DOCX 60 KB) [file 248_2023_2174_MOESM3_ESM.docx]

**Supplementary material**

EPILITHIC BACTERIAL ASSEMBLAGES ON SUBTIDAL ROCKY REEFS: VARIATION AMONG ALTERNATIVE HABITATS AT AMBIENT AND ENHANCED NUTRIENT LEVELS

Joseph Elsherbini^1^, Christopher Corzett^2^, Chiara Ravaglioli^3^, Laura Tamburello^4^, Martin Polz^1,5^, Fabio Bulleri^3*^

^1^ Department of Civil and Environmental Engineering, Massachusetts Institute of Technology, Cambridge, MA02138, USA

^2^ Molecular and Computational Biology Section, Department of Biological Sciences, University of Southern California, Los Angeles, CA 90089, USA

^3^Dipartimento di Biologia, Università di Pisa, CoNISMa, Via Derna 1, 56126 Pisa, Italy

^4^Department of Integrative Marine Ecology, Ischia Marine Centre, Stazione Zoologica Anton Dohrn, Punta San Pietro, 80077, Ischia, (Naples), Italy

^5^Centre for Microbiology and Environmental Systems Science, Djerassiplatz 1, 1130 Vienna, Austria

Figure S1. Relative abundance of ASVs assigned versus non assigned to different taxonomic levels
